# Case Report: Percutaneous catheter drainage in the treatment of amebic liver abscess with syphilis infections — a case study and literature review

**DOI:** 10.3389/fmed.2025.1620317

**Published:** 2025-08-13

**Authors:** Ting-Teng Yang, Han-Chuan Chuang, Wen-Chao Chen, Yu-Shan Hsieh

**Affiliations:** ^1^Division of Gastroenterology and Hepatology, Department of Internal Medicine, Taipei Medical University Hospital, Taipei, Taiwan; ^2^Division of Gastroenterology and Hepatology, Department of Internal Medicine, Lotung Poh-Ai Hospital, Yilan, County, Taiwan; ^3^Division of Infection Diseases, Department of Internal Medicine, Taipei Medical University Hospital, Taipei, Taiwan; ^4^School of Nursing, National Taipei University of Nursing and Health Sciences, Taipei, Taiwan; ^5^Department of Research, Taipei Medical University Hospital, Taipei, Taiwan

**Keywords:** percutaneous catheter drainage, liver abscesses, multiple infected, syphilis, amebic abscess

## Abstract

**Introduction:**

Amebic liver abscesses (ALAs) result from *Entamoeba histolytica*, a protozoan parasite transmitted through contaminated food or water. Diagnosis relies on imaging and serology, and treatment typically involves antibiotics such as metronidazole.

**Case presentation:**

Herein, we present a case of a 47-year-old man who presented with hepatitis B virus and syphilis infections during admission. Laboratory tests and computed tomography scan revealed a liver abscess. Percutaneous catheter drainage (PCD) was conducted on the day of admission. Positive amebiasis titers confirmed ALAs. Although culture of the purulent material from the abscess later showed no bacterial growth, ceftriaxone and metronidazole were kept empirically. The patient was discharged in stable clinical condition, and the drainage tube was removed 2 weeks after discharge.

**Conclusion:**

Our case demonstrated a scenario in which continuous PCD was initiated alongside traditional medical treatment in the risk group for ALAs and PCD complications. The symptoms were successfully relieved, and he recovered well without any complications.

## Introduction

Amebic liver abscesses (ALAs) are caused by *Entamoeba histolytica*, a protozoan parasite that is acquired via ingestion of food or water contaminated with human feces (1). In over 90% of patients, the trophozoites feed on intestinal tissue and bacteria without producing any symptoms. Nonetheless, in less than 1% of cases, the trophozoites penetrate the mucosa and reach the liver through the portal route, leading to the formation of liver abscesses (2). This disease is found almost exclusively in men (male: female > 10:1) (3–5). Most cases occur in middle-aged individuals, typically aged between 20 and 50 years (6). The highest rates of infection with *E. histolytica* are observed in India, Africa, Mexico, Central America, and South America (7). Previous studies have also shown a strong association between this disease and immunocompromised groups (risk group), such as patients with human immunodeficiency virus (HIV), diabetes, and syphilis (8).

Syphilis has also been recognized to involve the liver in the form of syphilitic hepatitis or hepatic disease, particularly in the secondary or tertiary stages of the disease. Although rare, liver involvement may alter the host’s hepatic immunity and contribute to the pathogenesis or severity of concomitant infections such as ALAs (9, 10).

Patients who have ALAs typically present with right upper quadrant (RUQ) pain and fever, while diarrhea and jaundice are less common. The onset is often subacute. A diagnosis usually is made via a combination of characteristic imaging and serologic findings. The treatment of ALAs usually requires antibiotics alone. The drug of choice is metronidazole, which has a cure rate of >90% (9). If there is a potential for drug resistance, other alternatives include tinidazole, ornidazole, and nitazoxanide (11–13).

A comprehensive review of the literature indicates that percutaneous drainage is typically indicated in two distinct clinical scenarios. The first scenario involves patients presenting with acute, severe, or fulminant disease. In these cases, drainage is undertaken urgently to arrest disease progression and prevent complications such as organ failure. Such abscesses have been described using various terms that underscore their aggressive nature, including “acute aggressive ALA,” “severe ALA,” and “fulminant ALA.” The second scenario pertains to patients who present later in the disease course with relatively mild symptoms but harbor large, persistent abscesses that fail to resolve with standard medical therapy. These cases are often referred with terms such as “drug-resistant ALA,” “refractory ALA,” or “chronic indolent ALA” (2).

To date, no consensus has been reached regarding whether drainage is necessary for ALAs, especially in patients with coexisting infections such as syphilis. In contrast, the treatment principles for pyogenic liver abscesses (PLAs) are well established and supported by a large body of literature (14–16). In the case of PLAs, treatment involves antibiotics targeting the causative organism coupled with drainage via a percutaneous catheter or needle aspiration. On the other hand, ALAs are treated with metronidazole, and only 15% of cases require percutaneous drainage (17). The causative agent of ALAs is *E. histolytica*, while PLAs are caused by bacteria such as *Klebsiella pneumoniae*, *Streptococcus milleri*, *Escherichia coli*, *Burkholderia pseudomallei*, *Staphylococcus aureus*, and *anaerobic bacteria* (18). Globally, ALAs are more common in low-middle income countries and middle-aged men (30–50 years old). In contrast, PLAs tend to occur more frequently in older patients (17). In addition, most patients become asymptomatic within 72–96 h of medical therapy. Drainage of ALAs is not necessarily imperative. Since no clinical benefit of drainage of ALAs has been shown, this procedure is thus not recommended as part of routine treatment (4, 19, 20). However, in these cases, drainage may alleviate patient pain without increasing infection risk, thereby offering frontline medical professionals additional treatment options for such patients. Herein, we present an interesting case of an ALA managed using a combined approach of percutaneous catheter drainage (PCD) and simultaneous antibiotic therapy. We also conducted a literature search on related research using PubMed and Google Scholar databases and search terms such as “amebic liver abscess,” “percutaneous catheter drainage,” and “syphilis.”

## Case presentation

A 47-year-old man without known systemic diseases presented to the emergency room with persistent right flank pain lasting a week. The pain characteristics were as follows: strangling, persistent, and visual analog scale score of 6 out of 10. The pain was located in the right posterior mid-back, and it could be relieved by leaning forward. He reported intermittent chillness but denied dysuria, hematuria, urinary frequency, diarrhea, abdominal pain, or fever. There was no change in the color of the urine or stool. He had visited the local medical department four times, but symptoms persisted. No recent travel history or mountain/water contact was reported. Physical examination revealed anicteric sclera, bilateral clear breath sounds, right upper quadrant abdomen tenderness, and right costovertebral angle tenderness.

**Figure 1:**
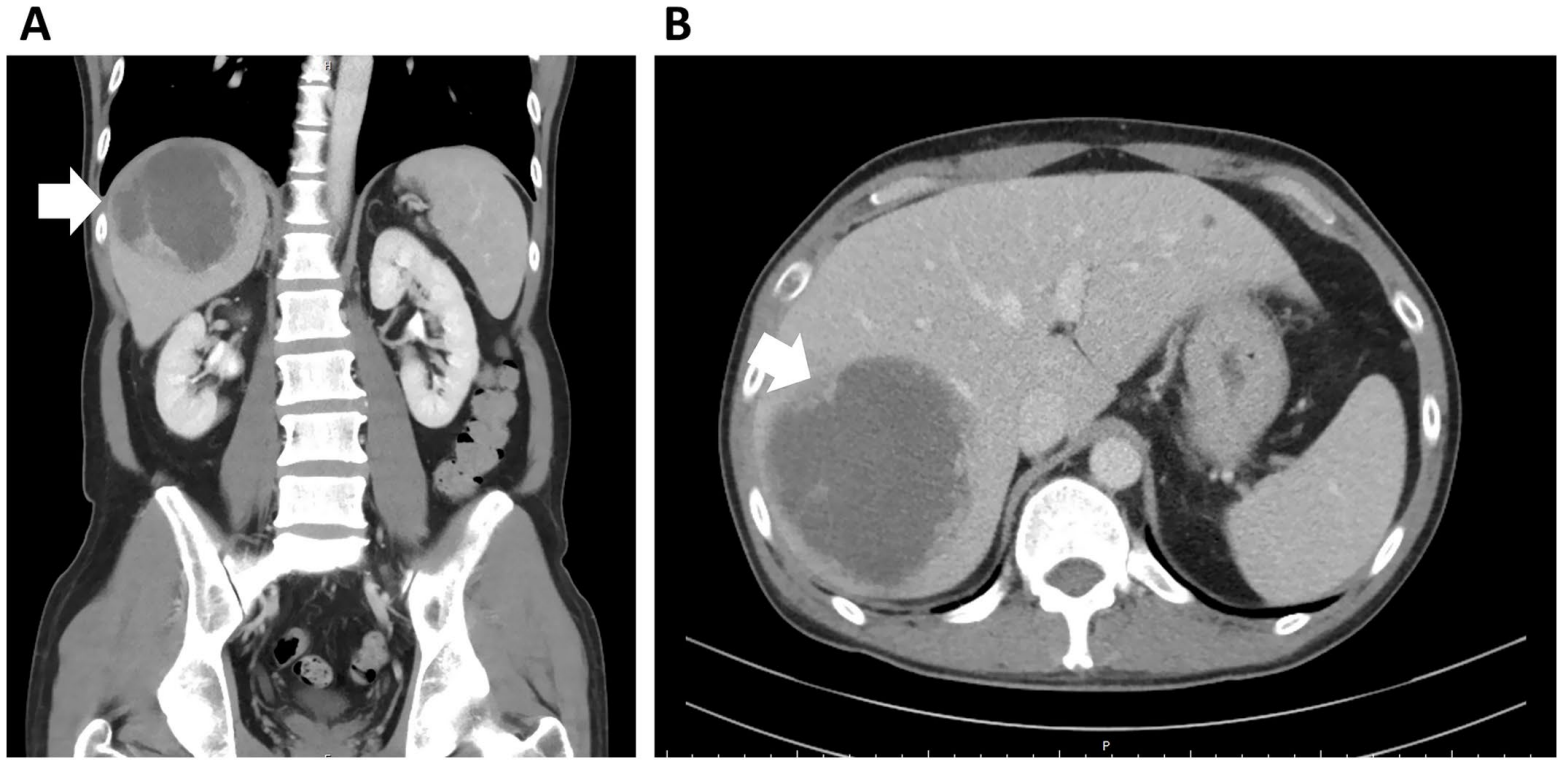
CT abdomen liver abscess examination. **(A)** A large abscess in the S7 segment of the liver was observed (white arrow). **(B)** Axial views, 8.9 cm x 8.0 cm abscess was observed (white arrow).

Laboratory tests revealed leukocytosis (white blood cell count: 31,330 /uL, reference: [4.00–11.00 /uL]) with a left shift (band form: 2%), an elevated C-reactive protein (CRP) level (23.79 mg/dL, reference: [<0.5 mg/dL]), impaired hepatic function (alanine aminotransferase [ALT]: 151 U/L, reference: [<41 U/L]), and hyponatremia (serum sodium level: 129 mEq/L, reference: [136–145 mEq/L]). Renal function testing and measurement of lactate and bilirubin levels were within normal limits. Pain levels also decreased after drainage (visual analog scale: 2–3). Abdominal CT scan revealed a liver abscess of 8.9 cm x 8.0 cm at S7, which is a solitary hypoattenuating lesion with rim enhancement (Figure 1).

Post-admission, empirical antibiotics including ceftriaxone (2,000 mg per day) and metronidazole (1,500 mg per day, intravenously) were administered from day 1 of admission. Given the abscess size exceeding 8 cm and the patient’s coexisting chronic infections, the clinical team decided to proceed with early PCD to reduce the risk of rupture and facilitate recovery. Immediate echo-guided PCD was performed for a suspected bacterial liver abscess by an interventional radiologist. The culture of purulent material from the abscess showed no bacterial growth. A stool culture of amoeba also showed no growth.

On the fifth day of antibiotic treatment, a positive serum amebiasis titer (indirect hemagglutination, IHA) confirmed the diagnosis of an ALA. A blood culture showed no bacterial growth. As a result, ceftriaxone was then de-escalated to cefmetazole at a dose of 3,000 mg per day on the fifth day of admission.

**Figure 2:**
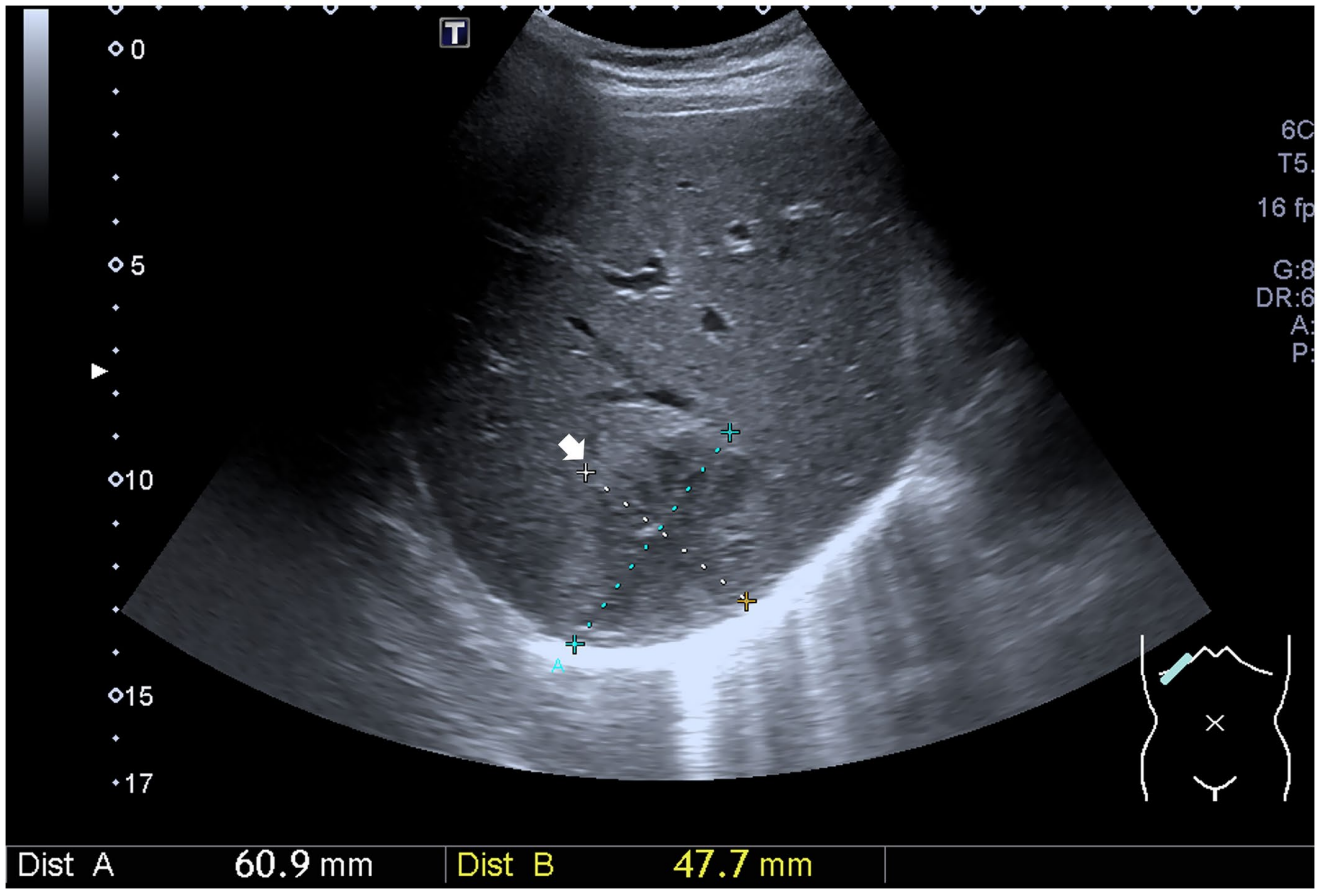
Ultrasound image shows a decreasing size of the liver abscess (white arrow).

**Figure 3:**
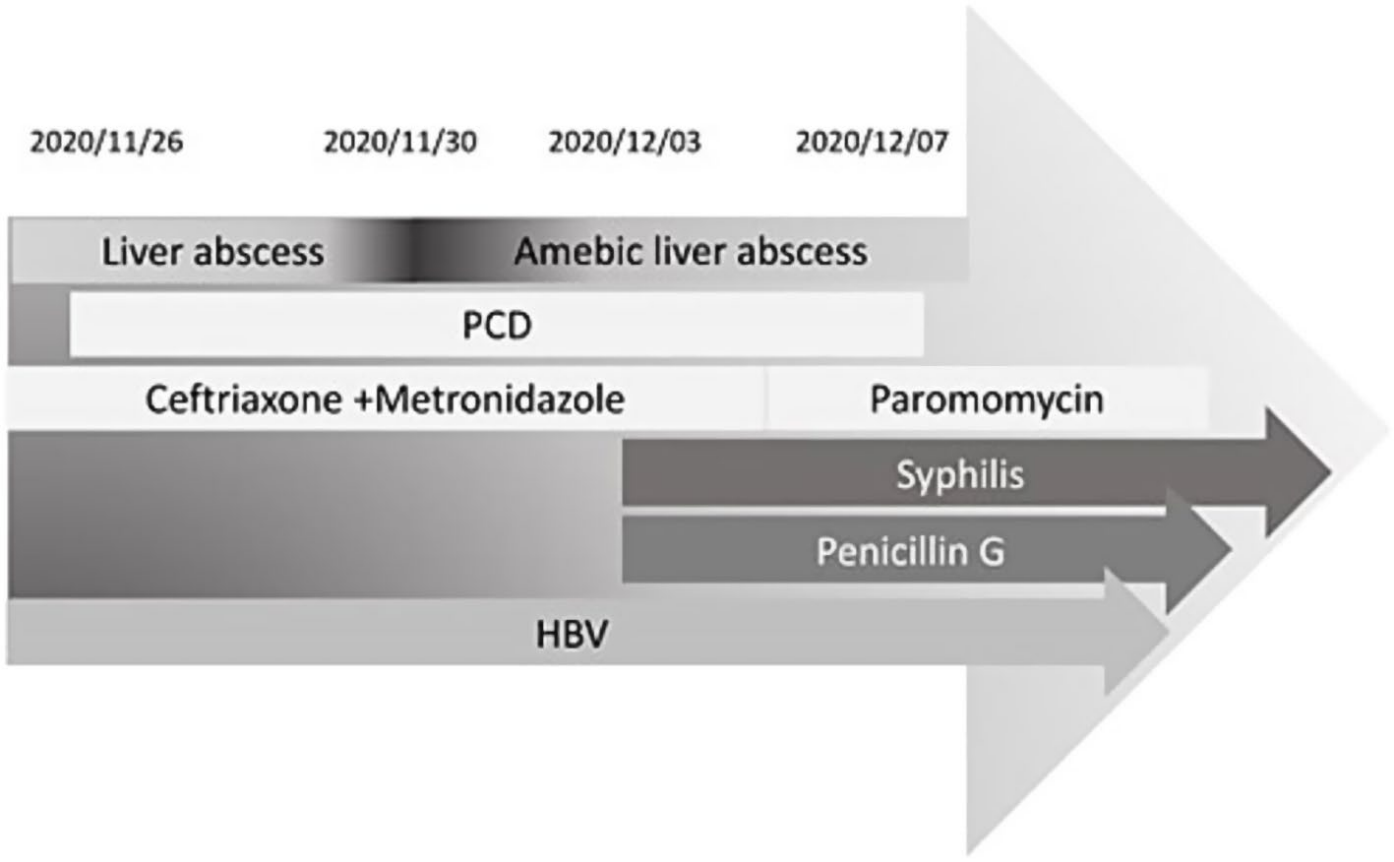
Timeline from November 2020 to December 2020. PCD, percutaneous catheter drainage; HBV, hepatitis B virus.

An abdominal sonogram performed on the seventh day of antibiotic treatment and 7 days after PCD revealed a decreasing size of the liver abscess to 6.1 cm x 4.8 cm (Figure 2). During detailed history taking, the patient disclosed engaging in high-risk sexual behavior. Infection was suspected to occur via special contact; hence, hepatitis B virus (HBV) and syphilis tests were checked, and both revealed positive results. No significant symptoms and signs of syphilis were noted, though. Penicillin-G (2.4 million units in a single dose) was then prescribed intramuscularly for syphilis on the 12th day of admission. The patient was discharged in stable clinical condition. Paromomycin (1,500 mg per day orally) was prescribed in the outpatient department. The drainage tube was removed 2 weeks after discharge due to minimal drainage output, and the patient recovered well. The clinical course is documented in Figure 3.

## Discussion

Our case demonstrated a rare scenario in which continuous PCD was initiated with traditional medical treatment. This case raises important questions regarding the necessity of drainage in cases of ALAs. Drainage is not customarily deemed mandatory as the clinical benefits were not previously evident, and routine treatment relies on antibiotics. Nevertheless, early PCD treatment was selected in this case, challenging the conventional approach.

There are some pros and cons of catheter drainage. Advantages of this technique include swift resolution of liver abscesses, rapid symptom relief, usage as an adjunct therapy for refractory cases, prevention of abscess rupture, and mitigation of vascular and biliary complications. Disadvantages of PCD involve catheter-related infection, bleeding, peri-catheter discomfort, prolonged hospitalization, and the inconvenience of catheter maintenance. Another concern with PCD is the potential development of fistulas from extended catheter placement, which could lead to trophozoites migrating to extrahepatic sites. Notwithstanding, to date, few studies or research have demonstrated such complications. The majority of fistulas typically existed before the placement of PCD, often as a result of the liver abscess itself. One study (21) advocated prompt PCD in all cases of ALAs that failed to respond to amoebicidal therapy. Another study (2) suggested that ALAs could be categorized into three forms: acute aggressive, subacute mild, and chronic indolent. The mild form of the disease can be easily cured with antibiotics alone. However, the other two forms often necessitate percutaneous drainage.

Although metronidazole therapy is typically the mainstay for treating ALAs, drainage is generally recommended in cases where the abscess exceeds 5 cm, is located in the left lobe, shows signs of impending rupture, or occurs in immunocompromised individuals (22). PCD is generally indicated for abscesses (1) left lobe liver abscess, (2) abscess with thin rim of hepatic parenchyma (<10 mm) around it, (3) multiple liver abscesses, (4) impending rupture recognized on imaging, and (5) non-response to medical therapy after 3–5 days, based on the existing literature and guidelines (23).

In our case, the patient presented with a large abscess (8.9 cm x 8.0 cm), persistent fever, and right upper quadrant pain. Imaging revealed a unilocular lesion near vascular structures. He was consistent with the “acute aggressive” form as described in the cited classification (2). Given the size and location and the patient’s immunocompromised status due to HBV and syphilis, the team opted for early PCD to prevent complications such as rupture or biliary obstruction. Although metronidazole was initiated upon admission, the clinical urgency prompted early intervention with drainage as an adjunct to medical therapy.

Percutaneous drainage of liver abscesses involves two methods: catheter drainage and needle aspiration. Most studies (24–27) have denoted that both percutaneous needle aspiration (PNA) and PCD are safe approaches for draining liver abscesses. PCD is considered superior to PNA because of its higher success rate, faster resolution of clinical signs, and a significant reduction in abscess cavity size. However, for some successfully medically treated patients, the outcomes of PNA are comparable to those of PCD. One study (28) revealed similar results, indicating that both procedures are equally efficacious in the management of liver abscesses.

To date, the consensus regarding drainage treatment for ALAs remains unclear. In immunocompromised hosts, such as patients with diabetes, cancer, HIV, syphilis, HBV, or cirrhosis, and those being treated with immunosuppressants, percutaneous drainage of liver abscesses (pyogenic or amebic) may (29, 30) or may not pose a potential risk (31–34).

The majority of previous studies (29, 33, 34) have reported no discernible therapeutic difference or increased complication rate between immunocompromised patients and the general population. One study (34) even demonstrated that the causative pathogens in HBV carriers were similar to those in general patients with abscesses. Therefore, the treatment plans for liver abscesses in the general population can be applicable to HBV carriers. However, there is currently no consensus or guidelines regarding drainage treatment for ALAs in syphilis. Although the literature on syphilis and liver abscesses is scarce, reports of syphilitic hepatitis and hepatic disease suggest that *Treponema pallidum* infection can impact hepatic structure and function. It is plausible that such alterations may predispose patients to secondary infections, including amebic liver abscesses (35–38).

The favorable outcome of PCD in this case can be attributed to multiple clinical and procedural factors. First, the patient presented with a solitary, unilocular abscess larger than 8 cm, which is a size associated with an increased risk of rupture and a mass effect on adjacent hepatic structures. Second, early initiation of PCD, within admission, allowed for the timely decompression of the abscess and the alleviation of systemic inflammatory response. Third, the absence of multilocation or septations, as revealed by imaging, facilitated complete drainage through catheterization. Moreover, the patient’s immunocompromised status, including co-infection with HBV and syphilis, necessitated more aggressive and timely intervention to prevent further deterioration. Together, these factors contributed to the rapid clinical improvement and resolution of the abscess.

Given that the risk groups for infection may include patients with chronic diseases, those with specific infectious diseases, and even the elderly, the patient’s medical history and medication history should be considered when determining the treatment approach for ALAs, making therapeutic decision-making for ALAs quite challenging. This is especially true when patients present with emergency symptoms (high fever, sepsis, severe abdominal pain, etc.), and the clinician’s decisions must simultaneously consider factors that could lead to adverse sequelae.

## Conclusion

Our case demonstrated a scenario in which continuous PCD was initiated with traditional medical treatment in the risk group in ALAs and PCD complications. The symptoms were successfully relieved, and he recovered well without any complications, even in the risk group.

Further research is warranted to establish clear guidelines on when drainage is beneficial in cases of ALAs, and factors such as the etiology, patient demographics, disease activity, progressing risks, and other risk factors should be considered. The presented discussion encourages a comprehensive understanding of ALAs and emphasizes the importance of individualized patient care. We shared this case to provide frontline medical professionals with expanded treatment options when managing similar patients.

## Data Availability

The raw data supporting the conclusions of this article will be made available by the authors, without undue reservation.
